# Prey items and predation behavior of killer whales (*Orcinus orca*) in Nunavut, Canada based on Inuit hunter interviews

**DOI:** 10.1186/2046-9063-8-3

**Published:** 2012-01-30

**Authors:** Steven H Ferguson, Jeff W Higdon, Kristin H Westdal

**Affiliations:** 1 Fisheries and Oceans Canada, Central and Arctic Region, 501 University Crescent, Winnipeg, Manitoba R3T 2N6 Canada; 2 Department of Environment and Geography, University of Manitoba, 501 University Crescent, Winnipeg, Manitoba R3T 2N6 Canada; 3 Higdon Wildlife Consulting, 45 Pilgrim Avenue, Winnipeg, Manitoba R2M 0L3 Canada; 4 Oceans North Canada, 515-70 Arthur Street, Winnipeg, Manitoba R3B 1G7 Canada

**Keywords:** beluga whales, bowhead whales, group size, hunting behaviour, narwhal whales, predator-prey relations, prey capture techniques, Traditional Ecological Knowledge, seals, walrus

## Abstract

**Background:**

Killer whales (*Orcinus orca*) are the most widely distributed cetacean, occurring in all oceans worldwide, and within ocean regions different ecotypes are defined based on prey preferences. Prey items are largely unknown in the eastern Canadian Arctic and therefore we conducted a survey of Inuit Traditional Ecological Knowledge (TEK) to provide information on the feeding ecology of killer whales. We compiled Inuit observations on killer whales and their prey items via 105 semi-directed interviews conducted in 11 eastern Nunavut communities (Kivalliq and Qikiqtaaluk regions) from 2007-2010.

**Results:**

Results detail local knowledge of killer whale prey items, hunting behaviour, prey responses, distribution of predation events, and prey capture techniques. Inuit TEK and published literature agree that killer whales at times eat only certain parts of prey, particularly of large whales, that attacks on large whales entail relatively small groups of killer whales, and that they hunt cooperatively. Inuit observations suggest that there is little prey specialization beyond marine mammals and there are no definitive observations of fish in the diet. Inuit hunters and elders also documented the use of sea ice and shallow water as prey refugia.

**Conclusions:**

By combining TEK and scientific approaches we provide a more holistic view of killer whale predation in the eastern Canadian Arctic relevant to management and policy. Continuing the long-term relationship between scientists and hunters will provide for successful knowledge integration and has resulted in considerable improvement in understanding of killer whale ecology relevant to management of prey species. Combining scientists and Inuit knowledge will assist in northerners adapting to the restructuring of the Arctic marine ecosystem associated with warming and loss of sea ice.

## Background

In recent years there has been significant interest in the role of killer whale (*Orcinus orca*) predation in shaping marine ecosystems and regulating prey populations [1]. Killer whales are widespread in world oceans and are the top predator in all regions where they occur [2-4]. The species consumes a wide variety of prey items, ranging from small schooling fish to large baleen whales [5], and there has been considerable debate over the role of killer whale predation in trophic cascades and prey species dynamics [6-8]. Killer whale predation can limit small prey populations [9-11], but more information on the species and number of prey consumed is needed to better address this issue [12]. Researchers have determined that in many areas killer whales with different and largely non-overlapping foraging specializations can co-exist. Examples include the coastal temperate northeast Pacific, where the transient ecotype feeds primarily if not exclusively on marine mammals, and the resident ecotype eats fish [13,14]. Four to five different ecotypes with different prey preferences have been identified in Antarctic waters [15-17]. Similar patterns have been identified for the northeast Atlantic [18], and to some extent for the eastern Tropical Pacific and southern Indian Oceans [19].

**Figure 1:**
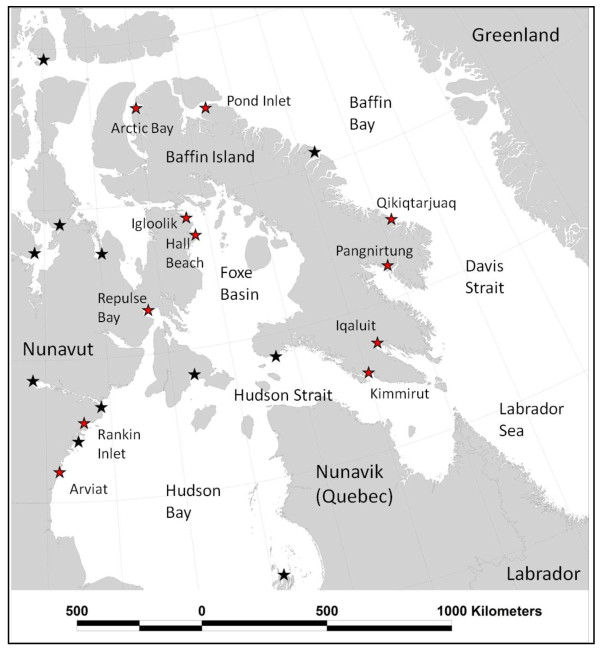
**Map of Nunavut showing location of communities that participated in the interviews about killer whale predators and their prey**.

In most other regions of the world, little is known about the ecology, life-history, and population status of killer whales [4]. One of these areas is the eastern Canadian Arctic (Figure 1), where there has been little directed research on killer whales until recently [20,21]. Killer whales were historically present in Davis Strait and Baffin Bay during the ice-free season, where they were occasionally reported in whaling logbooks in the 1800s [22]. The species appears to have recently colonized the Hudson Bay region [12], where sightings are increasing, possibly in response to declining summer sea ice distribution [23]. Killer whales are seasonal visitors to high latitude regions and sightings peak during the summer months (August and September) [24]. They have been observed killing and consuming other marine mammals, including both cetaceans (beluga (*Delphinapterus leucas*), narwhal (*Monodon monoceros*), bowhead (*Balaena mysticetus*)) and phocid seals (e.g., ringed (*Pusa hispida*) and bearded (*Erignathus barbatus*) seals [12,22,24,25]). These species are important to Inuit cultural and socio-economic well-being, and local hunters have expressed concern over increasing predation pressure on narwhal, beluga, and other Arctic marine mammals [12,26-28].

Traditional Ecological Knowledge (TEK) is being increasingly used in Nunavut [29-32], providing a long temporal record of rare species. Both Inuit and research observations of killer whale hunting and feeding can be scarce and hunting behaviour cryptic and hard to interpret. Observations of predation are rarely acquired by scientists, but Inuit hunters spend significant amounts of time on these waters hunting, fishing, boating, and observing marine mammals. We interviewed Inuit hunters and elders in Nunavut to develop an extensive baseline knowledge of Arctic killer whales. We collected information via 105 semi-directed interviews [33,34], including questions on killer whale prey items, hunting techniques, prey avoidance behaviour, and consumption patterns. Interviewees also provided information on killer whale abundance, distribution, and movements, which is summarized elsewhere [12,24].

## Methods

We used semi-directed interviews with Inuit hunters to document spatial and textual information on killer whales and their prey. This method provides a way to collect TEK in an open and flexible manner and avoids the rigidity of questionnaires [33,34]. A list of questions was developed in advance (Appendix 1), based on knowledge gaps of killer whale information (e.g., prey items, hunting behaviour), but interviews remained open-ended and each interviewee was given the option to elaborate on matters that were important to them. Interviewees do not always address every topic, but the approach also allows the interviewee to provide important information not anticipated by the researcher. Note that quotes are usually via the interpreter and not a direct quotation from the interviewee although some were in English. The research protocol was approved by the Office of Research Services, University of Manitoba (i.e., ethics approval) and the Nunavut Research Institute (NRI), and interviews were conducted with the consent and assistance of local Hunters and Trappers Organizations/Associations (HTO/HTA) in each community.

Communities (Table 1 Figure 1) were chosen to provide a wide representation throughout the Hudson Bay and Baffin Island regions of Nunavut and with consideration of historic and recent killer whale observations [24,27] and logistical constraints. Interviews were conducted with the aid of a local interpreter, with most conducted in Inuktitut (some in English). Spatial information (hunting and traveling locations, outpost camps, killer whale sightings, migration routes, etc.) was recorded on maps copied from the Nunavut Land Use Study [35]. Interviewees were identified using a reputational sampling method [36] where the local HTO in each community provided a list of potential interviewees, augmented with other participants identified by the interpreters or other interviewees (i.e., snowball technique [37]). Our research protocol allowed participants to remain anonymous or have their names acknowledged as a source of information, but stipulated that their names not be attached to any specific comments. All participants, that chose to be acknowledged, are listed in Appendix 2.

**Table 1:** Summary of eleven Nunavut communities visited to collect Inuit traditional ecological knowledge (TEK) of killer whales predation, with dates of visit, number of semi-directed interviews conducted, and region used for analyses.

Community	Date visited	Region	No. interviews
Repulse Bay	July-August 2007	Hudson Bay	17

Igloolik	February-March 2008	Foxe Basin	16

Hall Beach	February-March 2008	Foxe Basin	7

Rankin Inlet	March 2008	Hudson Bay	10

Arviat	March 2008	Hudson Bay	5

Pangnirtung	January 2009	South Baffin Island	11

Kimmirut	February 2009	South Baffin Island	5

Arctic Bay	April 2009	North Baffin Island	11

Iqaluit	April-May 2009	South Baffin Island	7

Pond Inlet	March 2010	North Baffin Island	8

Qikiqtarjuaq	March 2010	North Baffin Island	8

Total	July 2007-March 2010		105

A mix of qualitative and quantitative survey data on killer whales was collected and analyzed. Here we focus on the predation information only, without any specific focus on the information related to abundance, group sizes, spatiotemporal distribution, migrations and movements, etc. (data on file), although we give brief mention of these subjects where appropriate. Qualitative data were analyzed using an interpretive approach to connect ideas and categorize results [38], where results are grouped into related categories (e.g., specific prey items) and patterns summarized. Results were summarized within and between communities and four different regions (Table 1), and across Nunavut as a whole. All individual sighting reports were added to a larger killer whale sightings database [27], and observations from nine communities (pre-2010 interviews) were included in a recent analysis of the database, including predation observations [24] (also see [11,12] regarding communities in the Hudson Bay region).

## Results

We conducted 105 semi-directed interviews in 11 Nunavut communities between July 2007 and March 2010 (Table 1, Figure 1) (5-17 interviews per community: mean 9.5). Most interviewees were male (91%, one female interviewee in Arctic Bay, Arviat, and Rankin Inlet, two in Hall Beach, Pangnirtung, and Pond Inlet). We made an effort to interview older hunters, and 71 of 89 interviewees who provided age information were born in the 1950s or earlier. Younger hunters were interviewed on occasion, but only one interviewee was under 30. Most participants were active hunters or formerly active hunters (full-time harvesters) when they were younger, and most interviewees have spent a considerable amount of time hunting, fishing and boating throughout their lives (data on file). Nearly all participants (97%) had seen killer whales at least once, although not necessarily in the vicinity of their current community. Informants provided information on the seasonal and spatial distribution of killer whales in Nunavut, in addition to information on movements, migrations, and relative abundance. This information is presented in detail elsewhere [24].

### Marine mammal prey items

**Figure 2:**
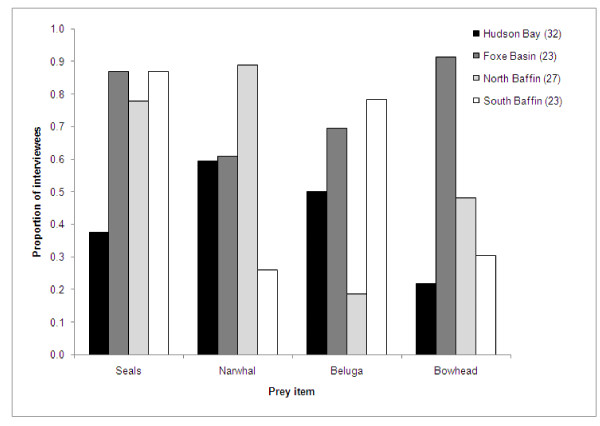
**Proportion of interviewees in each region reporting different marine mammal species as killer whale prey items**.

Participants in all communities provided extensive information on killer whale prey items. Inuit throughout Nunavut reported that marine mammals are the main prey items, and that all species of the phocid seals and the most commonly occurring cetaceans are consumed. Most interviewees reported multiple prey items, and five (two in Repulse Bay, three in Igloolik) referred to killer whales as the "wolves of the sea", or being "like wolves". Killer whales were reported to "eat whatever they can catch", "eat anything", and similar remarks by 14 interviewees (seven in Foxe Basin, three each in Repulse Bay and south Baffin, and one in north Baffin). Phocid seals (mainly ringed seals, but also harp (*Pagophilus groenlandicus*), bearded, and hooded (*Crystophora cristata*) seals, in descending order of frequency) were most commonly reported as prey (73 interviewees, 70% of total), followed by narwhal (63 interviewees, 60%), beluga (55, 52%) and bowhead whales (48, 46%). All communities and regions indicated similar marine mammal prey items, but the proportions of interviewees indicating each species varied by region (Figure 2).

**Figure 3:**
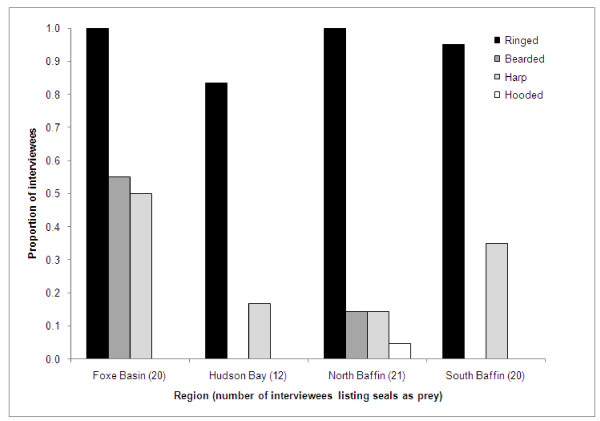
**Proportion of Inuit interviewees in different Nunavut regions reporting phocid seals as killer whale prey, by seal species**.

Reports of bowhead whales as a prey item were non-randomly distributed across regions (chi-square test, χ2 = 11.00, df = 3, P = 0.012), and concentrated in Foxe Basin (21 of 23 interviewees, 44% of the total interviewees that reported this species as prey). Bowhead predation was reported in the other three regions, but by fewer interviewees. Reports of narwhal as prey were also non-randomly distributed (χ2 = 11.22, df = 3, P = 0.011) and concentrated in the northern Baffin Island communities, with few south Baffin interviewees reporting this species as prey. Conversely, few north Baffin interviewees reported beluga as prey in comparison to the other three regions (χ2 = 7.63, df = 3, P = 0.055). Narwhal was also commonly reported as prey for the Hudson Bay region, particularly in Repulse Bay (16 of 17 interviewees). Reports of seals as prey items were evenly distributed across all four regions (χ2 = 2.89, df = 3, P = 0.409). One north Baffin interviewee (from Qikiqtarjuaq) also identified minke whales (*Balaenoptera acutorostrata*) as killer whale prey. Phocid seals were identified as prey by 73 (70%) interviewees, in all four regions (Figure 2). Most of these (70, 96%) identified ringed seals as a prey item, and this was the most commonly identified seal species in all four regions (Figure 3, ranging from 83% of the 12 Hudson Bay interviewees who listed "seals" to 100% of those in Foxe Basin and north Baffin). Harp seals were the second most commonly reported phocid prey, with 22 of the 73 interviewees (30%) identifying this species. Harp seals were identified as killer whale prey in all four regions, particularly in Foxe Basin and the south Baffin communities. Bearded seals were also mostly reported as a prey item by Foxe Basin interviewees (11 of 14 total reports). Hooded seals were reported as killer whale prey by one interviewee in Qikiqtarjuaq.

### Predation on fish

There is little evidence that Canadian Arctic killer whales eat fish [24], and interviewee responses suggested that predation on fish, if it does occur, is extremely rare (Table 2). Most interviewees (n = 79, 75%) did not mention or discuss fish as killer whale prey at all. Five interviewees noted that they did not know, or were not sure, if killer whales ate fish, and nine suggested they probably did, or might. Only four interviewees specifically stated that killer whales did not eat fish or that they had never heard of it occurring. Seven interviewees stated that killer whales did eat fish (five from Foxe Basin, two of which were in reference to the Pond Inlet area; and two from southern Baffin Island). However, none of these seven interviewees indicated that they had observed predation on fish first-hand.

**Table 2:** Summary of Inuit interviewee information and responses regarding killer whale predation on fish in Nunavut waters.

		Summary of interviewee responses about killer whales eating fish				
**Region**	**Community**	**No mention**	**Doesn't know, not sure**	**Maybe, probably**	**No, do not eat fish**	**Yes, do eat fish**

Hudson Bay	Repulse Bay (17)	17				

	Rankin Inlet (10)	9		1		

	Arviat (5)	5				

Foxe Basin	Igloolik (16)	5	1	4	2	4

	Hall Beach (7)	1	2	1	2	1

North Baffin Island	Arctic Bay (11)	9	2			

	Pond Inlet (8)	8				

	Qikiqtarjuaq (8)	8				

South Baffin Island	Kimmirut (5)	5				

	Iqaluit (7)	4		2		1

	Pangnirtung (11)	9		1		1

Total		79	5	9	4	7

### Killer whale hunting techniques

Interviewees provided extensive discourse of the hunting behaviour of killer whales, and described the methods used to hunt bowhead, narwhal, beluga, and seals.

#### Bowhead whales

In total, 35 interviewees provided information on the methods killer whales use to attack and kill bowhead whales, including 17 who described first-hand observations (most from Igloolik, 10). The other 18 interviewees described killer whale attack behaviour from stories they had heard from others (Table 3). Foxe Basin interviewees recounted either direct observations or second-hand stories of 13 different killer whale attacks on bowhead whales (data on file), and provided most of the information on this species as a killer whale prey item. Eight Foxe Basin interviewees estimated the number of bowhead whales killed there each year ranged from 3 to 10. Killer whale attack behaviour is summarized in Table 3. Most interviewees provided similar descriptions of the techniques used when killer whales attack bowhead whales that included: (1) to drown prey by holding the whale underwater and/or covering the blowhole and (2) immobilize the prey prior to suffocation by biting and holding on to the front flippers and tail, ramming the whale to cause internal damage such as breaking ribs, and tearing chunks of flesh out of the living whale. Many interviewees described the cooperative nature of killer whale attacks, such that some whales would be on top of the bowhead, others biting the flippers and tail, while others rammed it. Two interviewees also described observations where several killer whales continued to circle the bowhead, to keep it from escaping, while the others attacked. One interviewee noted that smaller groups of killer whales occur in Foxe Basin, where they concentrate on smaller bowheads; but larger groups of killer whales occur in Arctic Bay where they are able to kill adult bowhead whales. There were 14 bowhead attack observations that included an estimate of killer whale group size, ranging from 1-30 (mean 6.2, median 3.5).

**Table 3:** Summary of Inuit interviewee information and observations of killer whale attacks on bowhead whales, and observations of dead bowhead whales that were killed by killer whales.

Observations and information on killer whale attacks on bowhead whales			Region		
	**Foxe Basin**	**Hudson Bay**	**North Baffin**	**South Baffin**	**Total**

Interviewees reporting first-hand observations	10	2	5	0	17

Interviewees reporting second-hand observations and stories	6	4	2	6	18

Interviewees providing descriptions of attacks and attack methods	10	4	3	4	21

Killer whales:					

...circle the bowhead, to keep it from escaping while others attack	1		1		2

...hold the whale underwater and/or cover the blowhole, to drown it	8	2	2		12

...bite and hold on to the whale by the front flippers and/or tail flukes	5		1	2	8

...ram the whale in the side "to break ribs", and tear chunks out of the belly	5	2	2	4	13

Interviewees reporting dead bowhead whales that were killed by killer whales	16	1	5		22

Interviewees providing descriptions of dead bowheads	9		2		11

Hunters find dead bowhead whales:					

...with killer whale teeth marks on the flippers, tail, belly, and baleen	3		2		5

...with large chunks torn out and their bellies ripped open	5		2		7

...that have broken bones, busted ribs	1				1

...that are fresh kills, still warm	3				3

...that are fresh enough for meat and muktuk to be collected from	2				2

...that were killed for fun and not eaten, wasted by the killer whales	4				4

...that are being scavenged by polar bears	1				1

Inuit hunters occasionally found dead bowhead whales that were killed by killer whales, and this was noted by 22 interviewees. Reports were again mostly from Foxe Basin (Table 3), but dead bowhead whales were also reported from Hudson Bay (Repulse Bay) and northern Baffin Island (all three communities). Inuit assumed killer whales were responsible based on the external condition of the carcass (bite marks, chunks removed, evidence of internal injury such as broken ribs). Fifteen Foxe Basin interviewees identified locations of dead bowhead whales that had been killed by killer whales. Foxe Basin hunters reported finding from 3 to 5 dead bowhead whales in a single summer, with higher numbers in some years. A total of 32 observations of dead bowheads were reported for the region (including one in Lyon Inlet), and after correcting for overlapping reports, a minimum of 22 different dead bowheads were documented (with 8 found in 1999; data on file). In many cases, these whales were not eaten, or very little has been consumed (see below). In 1999, one was fresh enough for local Inuit to use as indicated by an interviewee in each of Igloolik and Hall Beach (Table 3).

#### Narwhal

Attacks on narwhal were observed by 24 interviewees, and four others reported stories they had heard from others. Five Foxe Basin interviewees had observed attacks, although most occurred in other areas (Table 4). Four Hudson Bay interviewees (all Repulse Bay) had observed attacks in their general area. Most observations (13) were reported by interviewees in north Baffin communities. Fourteen interviewees provided descriptive information on the methods used to kill narwhal. Prior to starting the attack, killer whales often herded the narwhal to a suitable location with deep enough water (see below), and also circled the group of whales to keep them stationary. Two interviewees noted that killer whales appear to tire the narwhal out prior to commencing an attack.

**Table 4:** Summary of Inuit interviewee information and observations of killer whale attacks on narwhals, and observations of dead narwhals that were killed by killer whales.

Observations and information on killer whale attacks on narwhals			Region		
	Foxe Basin*	**Hudson Bay**	**North Baffin**	**South Baffin**	**Total**

Interviewees reporting first-hand observations	5	4	13	2	24

Interviewees reporting second-hand observations and stories		1	3		4

Interviewees providing descriptions of attacks and attack methods	3		9	2	14

Killer whales:					

...herd narwhal to a suitable location with deep enough water, and circle to keep them stationary and prevent their escape	2		1		3

...tire out the narwhal prior to commencing an attack		1	1		2

...drown narwhal			2		2

...ram narwhal "to break their ribs"	1		5		6

...bite narwhal in the middle of the body, carry them in their mouth	2		3	1	6

...throw narwhal into the air, hit them with their tail and play with them, tear them apart and throw the pieces around	1		4	1	6

...leave lots of oil and scraps of blubber on the water	1	1	2		4

Interviewees reporting dead narwhals that were killed by killer whales	3	7	7		17

Interviewees providing descriptions of dead narwhals	2	6	6		14

Hunters find dead narwhals:					

...that are all busted up, with broken ribs	2	1	3		6

...that are covered with bite marks, with chunks and pieces missing		5	3		8

...that are killed but not eaten, killed for fun		2	1		3

...that are being scavenged by birds			1		1

...that meat and maqtaq can be collected from			3		3

* five Foxe Basin interviewees observed attacks on narwhal, but most occurred in other areas (Admiralty Inlet, 1; Pond Inlet, 1; Lyon Inlet, 2)

Interviewees reported killer whales ramming narwhal to "break their ribs", and killer whales will "play with" the narwhal, or pieces of them, and throw them around. One interviewee provided a second-hand description of killer whales "playing soccer" with the narwhal, and another observed killer whales killing narwhal by slapping them with their tail. Interviewees had observed killer whales biting narwhal, and sometimes carrying a narwhal in their mouth after biting them in the middle of the body. One Pond Inlet interviewee described two killer whales biting a narwhal and pulling it apart, leaving the head and tail behind and taking the "meat in the middle". Two interviewees reported that killer whales will also drown narwhal. One Igloolik interviewee described an attack in Admiralty Inlet, where the killer whales herded the narwhal close to shore, and then the smaller killer whales would come close to the shore to grab a narwhal and then head back out into deeper water, where the larger killer whales stayed. Participants in Repulse Bay noted that when they are stalking prey, killer whales will slow down, move very deliberately, and remain as quiet as possible in order to reduce the wake and sound produced by their dorsal fin moving through the water. When they are close to their prey they pick up speed.

Dead narwhals were often found with crushed abdomens and broken ribs, bite marks, and with pieces missing, and this was reported by 17 interviewees (Table 4). Two North Baffin hunters reported collecting *maqtaq *(skin and blubber) from narwhal that were killed by killer whales. An interviewee from Repulse Bay indicated that killer whales normally do not go after tusked narwhal, and another indicated that narwhal with tusks are never found floating dead. A Pond Inlet interviewee had observed killer whales going after female narwhal, but not males. He recounted a story that tusked narwhal had killed killer whales in the past; the narwhal pierced the killer whale with its tusk, and become stuck, with both animals dying as a result. Tusked narwhal may therefore represent a danger to killer whales; however, a Pangnirtung interviewee stated that they will attack narwhal with and without tusks, and two interviewees had observed killer whales kill tusked narwhal. An Arctic Bay hunter found a dead tusked narwhal covered with killer whale bite marks.

#### Beluga

Nineteen interviewees discussed killer whale attacks on beluga whales, with most from south Baffin (10, mostly (8) from Pangnirtung) (Table 5). Five interviewees told stories they had heard from others, the others recounted direct observations. Similar to attacks on narwhal, killer whales were reported to circle beluga before attacking, and killer whales have been observed ramming belugas with the presumed intent to cause internal damage that included broken ribs. Participants also reported that killer whales bit belugas in the mid-section, hold them in their mouth, and lift them out of the water, and will also toss them around. Predation events are often accompanied by oil slicks and scraps of blubber, and one interviewee noted gulls swarming around an attack site. A Pangnirtung interviewee told of a story he heard, where in the 1950s a person watched a killer whale eating a beluga that was still alive and moving. Seven interviewees noted that hunters often see beluga scraps (skin and blubber) floating in the water after killer whale attacks, and carcasses were sometimes found. One Pangnirtung interviewee saw a dead beluga after it had been hit and killed, the blubber was missing and the ribs were broken on both sides. Another noted that killer whales would sometimes kill "hundreds" of belugas and not eat them all. When the killer whales had left the kill site, Inuit would collect the *maqtaq *from the numerous dead belugas.

**Table 5:** Summary of Inuit interviewee information and observations of killer whale attacks on beluga whales, and observations of dead beluga whales that were killed by killer whales.

Observations and information on killer whale attacks on beluga whales			Region		
	**Foxe Basin**	**Hudson Bay**	**North Baffin**	**South Baffin**	**Total**

Interviewees reporting first-hand observations	4	3	1	7	15

Interviewees reporting second-hand observations and stories		3		3	6

Interviewees providing descriptions of attacks and attack methods	2	5	1	10	18

Killer whales:					

...circle the belugas before commencing an attack		1			1

...splash a lot when hunting belugas				1	1

...ram beluga whales in the side, "to break their ribs"			1	1	2

...toss belugas around, throw them in the air				3	3

...bite belugas in the midsection, lift them out of the water, carry them around	2			4	6

...leave lots of blubber scraps with bite marks, and oil slicks on the water		4		4	8

...kill beluga whales for fun, play with them, and sometimes do not eat them				2	2

...prefer the meat, and strip the blubber off the belugas				2	2

...will start to eat belugas when they are still alive				1	1

...sometimes eat belugas underwater, big pieces of blubber float to the surface		1			1

...leave blubber and dead whales behind that Inuit gather for food				1	1

Interviewees reporting dead belugas (or parts) that were killed by killer whales	2	1		4	7

Interviewees providing descriptions of dead belugas	2	1		4	7

Hunters find:					

...dead belugas with their ribs busted				1	1

...dead belugas with the blubber missing				1	1

...dead belugas covered in bite marks				1	1

...belugas that the killer whales have killed and left, wasting them				1	1

...scraps of blubber and maktak, all chewed up, floating in the water	2	1		4	7

#### Phocid seals

Twelve interviewees reported observations of attempted or successful predation on seals (ringed, harp and bearded seals), dominated by observations from south Baffin and Foxe Basin (Table 6). Four interviewees also noted second-hand observations and stories of killer whales attacking ringed and harp seals. Two north Baffin interviewees had observed killer whales slapping live ringed seals in the air with their tails, and four interviewees discussed the methods killer whales use to wash seals off small ice floes (reported for ringed and harp seals). Only two interviewees reported finding dead seals: a ringed seal in Foxe Basin (no additional details provided), and a harp seal (Iqaluit interviewee) that was found half eaten, by either a killer whale or a Greenland shark (*Somniosus microcephalus*).

**Table 6:** Summary of Inuit interviewee information and observations of killer whale attacks on phocid seals, and observations of dead seals that were killed by killer whales.

Observations and information on killer whale attacks on phocid seals			Region		
	**Foxe Basin**	**Hudson Bay**	**North Baffin**	**South Baffin**	**Total**

Interviewees reporting first-hand observations	4	1	2	5	12

Interviewees reporting second-hand observations and stories		1		3	4

Interviewees providing descriptions of attacks and attack methods			2	4	6

Killer whales:					

...throw live ringed seals in the air with their tails			2		2

...drive harp seals in all directions when they are pursued				1	1

...wash seals (ringed and harp) off of ice floes				4	4

Interviewees reporting dead seals that were killed by killer whales	1		1		2

Interviewees providing descriptions of dead seals	1		1		2

Hunters find:					

...dead ringed seals with bite marks	1				1

...dead harp seals that are partially eaten			1		1

All four observations of washing seals from ice were from south Baffin communities (two each from Kimmirut and Pangnirtung). Killer whales will circle a piece of ice and use their tails to create waves, washing seals off into the water. Two provided reports based on stories from others, but one hunter in each community provided a first-hand observation. One Kimmirut interviewee noted that killer whales could create enough turbulence to make waves and try to knock seals off, no matter the thickness of the ice floe, and the other described this behaviour as an example of the intelligence killer whales exhibit when hunting.

### Prey behaviour in the presence of killer whales, predator avoidance behaviour

**Figure 4:**
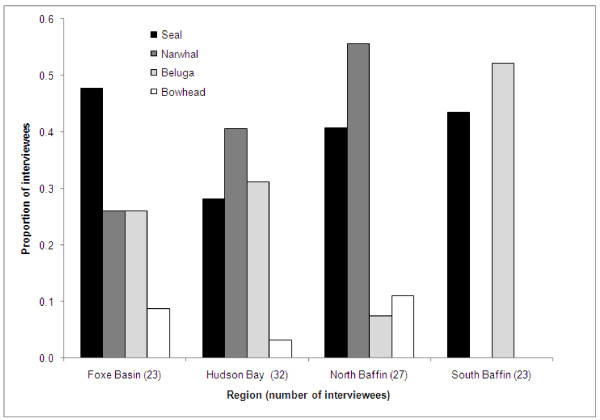
**Proportion of Inuit interviewees in different Nunavut regions reporting killer whale avoidance behaviour (going to shallow water) for different prey species (all phocid seal species combined)**.

When killer whales were present, all the different marine mammal prey species sought refuge in shallow waters close to shore as a predator avoidance technique. This behaviour is well-known among Inuit hunters and is known as '*aarlirijuk*' ("fear of killer whales") in the south Baffin dialect of Inuktitut ([39]; alternate spellings given by [40,41] are '*ardlingayuq*', '*ardlungaijuq*' or '*aarlungajut*'). The majority of interviewees (68%) reported that prey species will head to shallow waters and to the shoreline to avoid killer whales (with multiple species identified in some cases: 41 for seals, 34 for narwhal, 30 for beluga, and six for bowhead) (Figure 4). The proportion of interviewees identifying this behaviour was similar for the four regions, ranging from 61 to 70% of the total number interviewed. The prey species reported to exhibit this behaviour varied by region and community however, with narwhal observations predominating in North Baffin and Hudson Bay communities (primarily Arctic Bay and Repulse Bay, also Pond Inlet). Observations of seal avoidance behaviour were reported in all communities, and beluga observations were mainly reported by South Baffin interviewees (in all three communities).

Eleven interviewees noted the behaviour bowhead whales use to avoid killer whale predation (three each from Foxe Basin and Hudson Bay, five from north Baffin). Three reported that bowhead whales will "run away", and five indicated that they will run to the ice, where killer whales will not go, or into shallow waters, inlets and fjords. One interviewee (Repulse Bay) reported that bowhead whales will dive deep to avoid killer whales, and that they can stay down longer due to their larger lungs. A Rankin Inlet interviewee described a bowhead whale being chased by killer whales, and the bowhead was jumping out of the water. In one Igloolik observation (from August 2007), a killer whale was observed chasing after a bowhead and her calf. At one point during the chase, the entire bowhead calf was up out of the water. The interviewee thought that the cow was underneath holding the calf out of the water to protect it from the killer whale (the final outcome was not known).

Many interviewees had observed narwhal fleeing to shallow waters to avoid killer whales. One interviewee in Pond Inlet described seeing narwhal half-beached (the observation was also described by [42]), and one in Arctic Bay said there were once so many narwhal on shore that people could even touch them. Another Arctic Bay interviewee described a 2008 observation where so many narwhal fled into shallow water that it "looked liked waves on shore". A Pond Inlet interviewee reported that in 2008, he heard on the CB radio that killer whales were coming, and people headed to the area to hunt the narwhal that they knew would be gathering. Within 30 minutes the narwhal had gathered in the shallow water close to the shoreline. Killer whales are reported to prefer deep water, and heading to the shoreline is an effective anti-predator technique: an Arctic Bay hunter observed narwhal stay in the shallows long enough for the killer whales to give up and leave, and a Pond Inlet interviewee also observed this in the 1970s in Milne Inlet. Many killer whales came near, and were there for four days, but could not get the narwhal that were hiding in shallow waters and then left.

Three interviewees from Repulse Bay described differences in narwhal surfacing behaviour when killer whales were near. The narwhal will be more quiet than usual, and they stay lower in the water, just barely surfacing to breathe. One noted that narwhal will become aware of killer whales two to three days before they were seen by people. Another observed that narwhal swim faster when followed by killer whales than when hunted by boats. Narwhal will also use alternate travel routes when killer whales were near. One Repulse Bay hunter reported that narwhal would go to Wager Bay to avoid killer whales, although he had not seen this personally. Another hunter had observed narwhal in Wager Bay two years previously (2005), and noted that it was the first time he ever saw narwhal there, despite many trips to the area. Another Repulse Bay hunter indicated that narwhal have become less concentrated in certain predictable areas, and are more scattered in their distribution. Interviewees in Arctic Bay and Pond Inlet also described alternate routes and different staging areas that narwhal will use to avoid killer whales. As noted above, tusked narwhal were also reported to be able to defend themselves by stabbing or piercing an attacking killer whale, although this is likely to result in the death of both animals. An Igloolik interviewee suggested that belugas were arriving later in the season due to the presence of killer whales.

### Prey consumption by killer whales

Some participants noted that killer whales will sometimes kill and eat only a little of their prey, leaving the rest, and sometimes even not eat anything at all. This observation varied with prey type, with less eaten of larger prey (i.e., bowhead whales). However, even for the smaller cetaceans, only some parts were eaten as indicated by square *maqtaq *pieces being observed by Inuit after kills. Many interviewees noted what they termed as wastage by killer whales choosing the choicest parts. Eleven interviewees in four communities (Igloolik, 4; Pond Inlet, 1; Rankin Inlet, 3; Repulse Bay, 3) discussed killer whale wastage of prey (beluga, 4; bowhead, 3; narwhal, 1). Eight interviewees noted that killer whales sometimes "kill for fun", kill without eating, or "play" with wildlife. Three interviewees described wastage in a positive manner due to Inuit getting *maqtaq *from killed whales, while five comments were negative towards killer whales because they wasted food. Three interviewees also noted that killer whales appeared to prefer meat to blubber, as floating chunks of skin and blubber were often seen in large square pieces (and sometimes collected for human use).

## Discussion

Killer whales dominate marine ecosystems as top predators controlling top-down trophic interactions [6,43,44]. Thus, knowledge of food habits and predatory behaviour is key to conservation initiatives. Many Inuit interviewees noted that they did not have much knowledge about killer whales, in comparison to other marine mammals, because they are not hunted (or rarely hunted [27]). Despite these assertions, local Inuit have broad knowledge of killer whales as predators, and their observations represent a substantial addition to our knowledge base on this species in the Canadian Arctic, in terms of their distribution, prey selection and hunting behavior. This is the first dedicated survey of TEK on killer whales in Nunavut waters and both corroborates the results of previous investigators and provides new insights.

### Killer whale distribution and prey selection

Direct historical comparisons are difficult given differences in interview techniques and interviewees, but the results suggest an increase in observations of killer whale predation in recent years. Stewart et al. [36] collected TEK in the early 1990s on narwhal and beluga in four communities and solicited information on killer whale predation. Three communities overlap with our study (Arctic Bay, Igloolik and Hall Beach), allowing some comparison of results. Seven hunters from two Foxe Basin communities did not see any attacks on beluga or narwhal, and only one observed scars caused by an unsuccessful attack on beluga. In contrast, five of our 23 Foxe Basin interviewees observed attacks on narwhal (although most occurred in other areas such as Admiralty Inlet, see Table 4) and four saw attacks on beluga. In Arctic Bay Stewart et al. [36] interviewed six hunters for their knowledge of beluga and narwhal. One had seen a killer whale successfully attack a beluga, and another hunter observed scars on a beluga from an unsuccessful attack. One also saw six killer whales attacking a narwhal, and another observed a narwhal killed by a killer whale. Scars from unsuccessful attacks were also reported. In contrast, five of our 11 Arctic Bay interviewees had observed attacks on narwhal [36]), and three of these had also seen dead narwhals that were killed by killer whales. None reported observations of attacks on beluga whales.

Differences in prey species taken by killer whales observed in this study were likely due to non-random distribution of the prey species and/or regional selectivity by particular killer whale groups familiar with different regions. Such regional disparities have been noted by other researchers. Thomsen [45] summarized hunter observations of attacks on belugas and narwhals in West Greenland. In Disko Bay, five of 59 hunters had observed beluga being attacked, compared to four of 14 hunters from the Uummannaq District, one of 34 in the Upernavik District, and none of the 22 hunters interviewed in the Avanersuaq District. For narwhal, none of the 40 Disko Bay hunters or 24 Uummannaq District hunters had seen attacks, and they had been observed by only two of 33 Upernavik District hunters. Attacks were most frequently observed in northwest Greenland, where 15 of 31 Avanersuaq District hunters had seen them. These results, combined with ours, indicate that the north Baffin region of Nunavut (Arctic Bay, but also Pond Inlet) and northeast Baffin region of west Greenland are key locations with concentrated interactions between narwhal and killer whales. In addition to the north Baffin region, the Repulse Bay area was also a key killer whale-narwhal interaction area where four of 17 hunters observed attacks and seven had found dead whales.

Our TEK results support the expectation that killer whales eat multiple prey species, but appear to restrict their hunting to marine mammals in the selected study region. TEK results also suggests that killer whales in eastern Canadian Arctic waters do not feed on walrus, which contrasts with what is known about killer whale feeding habits in Arctic waters north of the Pacific Ocean [46,47].

Killer whales are apex predators with high energy requirements that can result in conflicts with human interests due to competition with commercial fisheries and interactions with endangered species. Therefore, there is a need to understand killer whale ecology, distribution, abundance, and evolutionary history to inform management and conservation. Interviewees in Foxe Basin (also see [39,48]) noted a link between a growing bowhead population and increasing killer whale presence. In the eastern Canadian Arctic most cetacean and phocid populations have increased since large-scale commercial whaling and sealing ended in the early to mid-1900s, and improved foraging conditions therefore exist for killer whales. Ferguson et al. [11] tested alternative predator-prey relationships within the Hudson Bay geographic region, where evidence suggested killer whales seasonally concentrate feeding activities on the large-bodied bowhead whale. Results indicated that killer whales feed on narwhal and beluga whales early and late in the ice-free season whereas feeding was focused on bowhead whales during summer. Using TEK estimates of prey mortality, Ferguson et al. [11] estimated that on average 57 bowhead (range 28-90), 112 narwhal (range 10-234), 174 beluga (range 12-326), and 117 seals (range 12-322) are likely removed annually from the region by killer whales. Thus, Inuit TEK can provide information necessary for modeling predation impact and thereby inform management and conservation decisions.

Killer whale ecotypes are morphologically recognizable forms that prefer different prey and as a result they have different foraging strategies, acoustic behaviour, and habitat preferences. Divergence likely originates through natural selection of an intelligent social animal that transmits cultural heritages within stable family groups [49]. A likely explanation for these differences in killer whale prey preferences would consider the profitability and risk of attack choices. Smaller marine mammal prey, such as seals, is not as profitable as baleen whales but presumably have lower risk of injury or death to the predator during an attack. At lower latitudes in less productive tropical waters, killer whales may have to be generalists [50,51] but at higher latitudes in more productive marine ecosystems killer whales typically specialize and the number of ecotypes appears to increase with increasing prey density. As many as three sympatric ecotypes have been reported in different communities (data on file) and this is a reasonable working hypothesis for the northwest Atlantic where fish-eaters have been observed in southern Greenland and Newfoundland and Labrador waters, shark-eaters may occur offshore, and marine mammal specialists enter the region seasonally. For the eastern Canadian Arctic, during the open-water season, the mammal-eating ecotype predominates. And it is this type of killer whale that Inuit are familiar with and it is the foraging behaviour of this type that we have documented using Inuit TEK.

Baleen whales, including bowheads, and Arctic odontocetes show strong seasonal migration patterns that include intensive feeding during spring through fall building up blubber stores for a winter season where feeding is rare. The eastern Canadian Arctic marine-mammal-eating killer whales also cycle seasonally in both summer-winter ranges and energy intake. In particular, arrival into the Arctic during the open-water season coincides with timing of peak pupping and calving that would presumably provide more easily accessible prey for killer whales. It has been estimated that higher latitude killer whale food intake rates during summer are approximately double the rest of the year [50]. Another study estimated that killer whales at high latitudes input 50% of their annual intake during a 90 day summer period [11]. As with many high-latitude large-bodied mammals, killer whales undergo an intense feeding period during summer followed by a relatively inactive negative energy period over winter. Inuit knowledge and observations have provided substantial information necessary to estimate seasonal variation in killer whale feeding needed to better assess the regulatory role of killer whale predation on their prey.

### Hunting behavior

A significant body of literature exists on the hunting behavior of killer whales and the responses of their prey. For example, numerous previous studies have described prey species fleeing to shallow waters to escape from killer whales [26,39,48,52-54]. Many of these sources also note the influence of killer whales on the movements of bowhead, narwhal and beluga whales [26,39,53-58]. The methods used by killer whales to kill marine mammal prey have also been described by interviewees in previous studies, as has the fact that killer whales do not always consume everything and leave pieces of skin and blubber behind which are sometimes collected by Inuit [26,36,39,45,59].

The results of our TEK study compliment the results of other studies and provides some new insights on foraging behavior in Nunavut waters. For example, Inuit observed killer whales attacking and killing large whales and then only consuming parts. Observations of killer whale eating baleen whales include ripping out only tongues and lips, the favored parts [17].

Most recorded attacks of killer whales on large whales are by relatively small groups of killer whales (1-5 [60]), as observed by Inuit [24]. Transient killer whales encountered around Southern California in April and May hunting gray whale calves involve groups of 2-4 individuals conducting the attacks [61]. Inuit have observed that killer whales are like canids (e.g., wolves *Canis lupus*) that hunt cooperatively and attack in a coordinated manner through communication when preying on ungulates [62,63]. Inuit observations also indicate that killer whales are cooperative pack hunters, like wolves, when pursuing fast or dangerous prey. Inuit observed that killer whales use prey capture techniques that utilize prior knowledge of landscape such as attacking prey before they can seek shallow water refuge [64-66]. Inuit observed that killer whales wash seals off of sea ice as observed in Antarctica [16,67].

Inuit ecological knowledge also provides new information that indicates that cetacean prey use sea ice as a refuge from killer whale predation, which has only been suggested in scientific literature [68-70]. Although noted for other regions [71,72], Inuit described Arctic killer whale foraging behaviour that involves a strategy of searching, finding prey, giving chase, and if unsuccessful, then waiting to see if prey leave refugia and following the landscape shoreline topography for opportunities to attack. An Igloolik interviewee observed a killer whale chasing a bowhead whale cow and calf, with the calf being held out of the water. The interviewee thought that the cow was underneath the calf holding it out of the water to protect it. Grey whales (*Eschrichtius robustus*) have been observed to respond to killer whale attacks by rolling at the surface to keep their ventral area from being exposed to killer whales below, and mothers have been observed to roll over and hold their calf above the water on their ventral surface to keep it away from killer whales [73,74]. To our knowledge, this report from Igloolik is the first time this behaviour has been noted for bowhead whales.

Marine mammal scientists have observed and described sophisticated techniques used by Antarctic Type B killer whales to hunt seals on ice floes [16,75]. Eastern Arctic killer whales are occasionally observed near light to moderate ice, and south Baffin interviewees described similar hunting behaviour with killer whales washing seals off ice floes (also see [24]). Although it has not been observed or described by scientists, Inuit have long recognized different hunting techniques killer whales use to catch seals on ice floes. In 1790, Fabricius noted reports from West Greenland hunters where killer whales worked in unison to lift an ice floe from underneath and cause harp seals to fall off into the water [76]. On northern Baffin Island, hunters have similarly seen killer whales surround an ice floe while one pushes up from underneath to break the ice and force seals into the water [55]. These observations add to a growing body of knowledge on the use of advanced and sophisticated hunting behaviour by killer whales.

In most cases the information provided in different communities and regions was complementary (e.g., prey items, prey avoidance behaviour, attack techniques). However, there is disagreement among opinions and/or observations of different behavior. Several Repulse Bay interviewees suggested that killer whale do not normally attack tusked narwhal and that only females are found dead. The same information has been reported previously [26], possibly from the same local experts. These observations are contrasted by those of other interviewees who indicate that tusked narwhal are killed on occasion. In northwest Greenland (Avanersuaq District), hunters have also observed killer whales attacking and killing both male and female narwhal [45]. A Pond Inlet interviewee reported a story of a narwhal piercing a killer whale with its tusk, suggesting that male narwhal are dangerous to attack. Rosing [77] described an observation from Greenland in December 1924 where killer whales were observed killing narwhal and one was seen jumping out of the water with a narwhal stuck to its side, with its tusk penetrating to the root straight through the killer whale. During interviews for the Igloolik Oral History project [78], an elder provided a story [79] of a dead killer whale that was found in the waters of *qaqqalik *(possibly near Kimmirut) that had a narwhal tusk pierced through its mouth.

## Conclusion

Inuit observations have provided a sound background of ecological information detailing Arctic killer whale feeding habits including hunting techniques that are specific to prey species and prey behavior associated with reducing the risk of killer whale attacks being successful. Such knowledge is critical to management and conservation efforts to ensure persistence of sustainable Inuit cultural hunting. Given the importance of long-term relationships between scientists and hunters for successful knowledge integration, this study provides an example of the potential for meaningful integration in short-term projects such as incorporating Inuit understanding of the ecology of killer whale predation in an assessment of the forms of active participation by TEK-holders in science development. We expect considerable improvement in knowledge of killer whale ecology relevant to management as scientists and Inuit combine forces to tackle a major conservation issue - the rapid shifting of the Arctic marine ecosystem structure associated with warming and loss of sea ice.

## Competing interests

The authors declare that they have no competing interests.

## Authors' contributions

SHF was the project manager responsible for conceiving, coordinating, and obtaining the funding to carry out the survey of Inuit knowledge and wrote the final draft of the manuscript. JWH carried out surveys in two communities, performed the statistical analysis, and wrote the first draft of the manuscript. KHW conducted 9 of the community surveys and helped write the manuscript. All authors participated in planning, coordinating, and conceiving the study design and have read and approved the final manuscript.

## Appendix 1

Inuit knowledge on killer whales

Part 1 - General information (Optional)

Name:

Age:

Community:

Years living in community:

How often do you go hunting or fishing (on the ocean, not land)?:

(Example: twice a week, on weekends, once every 2 weeks)

Is this more or less time than five year ago?: Ten years ago?

In a year, how many days do you spend on the water?:

How far do you travel during hunting trips?:

Part 2 - Killer whales

Have you seen killer whales in the past?:

Sightings:

Date (Year, Location Number of killer Behaviour** Comments month, etc) (Use map) whales

* use the provided map to mark the location of the sighting, and give each map sighting a unique number which will be listed on the table

** What were the whales doing? swimming direction, preying on other species, etc

How often are killer whales observed in this area (e.g., every year, only some years)?:

What time of year are killer whales around?:

Do killer whales stay for a while or do they show up briefly and then leave again?:

Do killer whales use the same areas each year? Which areas?

What travel routes do killer whales use?

Are killer whale numbers increasing?:

Decreasing?

Not changing?

How many killer whales do you think use this area?

What animals do killer whales eat?:

Have you seen killer whales attacking other species? If yes, which species?

Do you think killer whales are having a negative impact on other species? If yes, which other species?

Have you ever taken photos of killer whales? Do you know anyone else who has?:

## Appendix 2

We thank the following people for sharing their knowledge with us and facilitating the research, with communities and interviewees in alphabetical order:

Arctic Bay: M. Akumalik, A. Huges, N. Iqalukjuak, J. Kalluk, L. Kalluk, I. Kigutijuk, L. Koonark, I. Koonoo, K. Oyukuluk, S. Qaunaq, and I. Shooyook (interviewees); D. Koyukuluk (interpreter); Ikajutit HTO

Arviat: L. Angalik Sr., J. Kaludjak, P. Kaludjak, J. Karetak, and Rev. J. Muckpah (interviewees); Frank Nutarasungnik (interpreter), Arviat HTO

Hall Beach: A. Allianaq, S. Arnardjuak, D. Irqittuq, D. Issigaitok, S. Kaernerk, P. Pikuyak, and R. Siakuluk (interviewees); L. Ningmalik (interpreter); Hall Beach HTA

Iqaluit: I. Adamie, J. Adamie, C. Erkidguk, I. Inookee, A. Inookie, J. Kownirk, and A. Sata (interviewees); Adamie Inookie (interpreter); Amarok HTO

Igloolik: S. Allurut, S. Ammaq, A. Arnatsiaq, M. Arnatsiaq, E. Ipkarnak, D. Irngaut, H. Ittuksarjuat, A. Ivalu, J. Kopak, E. Kunuk, L. Makkik, C. Piugattuk, S. Qammanirq, A. Qrunnut, A. Ulayuruluk, and L. Uttak (interviewees); J. Kopak (interpreter); Igloolik HTA

Kimmirut: S. Akavak, T. Akavak, S. Aqpik, E. Padluq, and Q. Pudlat (interviewees); S. Aqpik (interpreter); Mayukalik HTA

Pangnirtung: M. Battye, J. Keakee, S. Keenainak, O. Kilabuk, P. Kilabuk, M. Kisa, E. Nashalik, P. Nauyuk, M. Noah, P. Quappik, and D. Veevee (interviewees); L. Kanayuk (interpreter); Pangnirtung HTA

Pond Inlet: P. Enooagak, L. Kadloo, P. Komangapik, T. Maktar, J. Muckpa, C. Nutorak, E. Panipakoocho, and J. Simonee (interviewees); T. Arnakallak and R. Soucie (interpreters); Mittimatalik HTA

Qikiqtarjuaq: L. Alikatuktuk, J. Alookie, J. Keyooktak, I. Kokseak, J. Newkingngak, T. Newkingnak, A. Kooneeliusie, and L. Nutaralak (interviewees); H. Olookie (interpreter); Nattivak HTA

Rankin Inlet: M. Innukshuk, P. Ipkorneak, H. Ittinuar, O. Ittinuar, J. Kabvitok, F. Kaput, N. Makayak, M. Tarparti, J. Tattuinee, and R. Tatty (interviewees); Norman Ford (interpreter); Kangiqliniq HTA

Repulse Bay: H. Aglukka, M. Akkuardjuk, L. Angogingoar, J. Ignerdjuk, J. Ivalutanar, S. Kidlapik, L. Kringayark, L. Malliki, Q. Malliki, S. Malliki, D. Milortok, L. Putulik, P. Sanertanut, A. Siatsiak, M. Tagornak, C. Tinashlu, and D. Tuktudjuk (interviewees); M. Tungalik (interpreter); Arviq HTO
